# Hand-carried ultrasound use in clinical nephrology: Case report

**DOI:** 10.1097/MD.0000000000004166

**Published:** 2016-07-29

**Authors:** Kathryn D. Winters, Stephanie Toth-Manikowski, Carol Martire, Tariq Shafi

**Affiliations:** a Department of Medicine; b Division of Nephrology, Department of Medicine, Johns Hopkins Bayview Medical Center; c Welch Center for Prevention, Epidemiology and Clinical Research, Johns Hopkins University, Baltimore, MD.

**Keywords:** acute kidney injury, cardiorenal syndrome, hand-carried ultrasound, hemodialysis, jugular venous pressure

## Abstract

**Background:**

Correctly assessing and managing volume status are critical elements of daily care for patients managed by nephrologists. However, intravascular volume is difficult to assess by physical examination alone.

**Methods:**

We present vignettes illustrating the potential for using hand-carried ultrasound (HCU) to improve volume assessment in common clinical scenarios faced by the renal consultant in the hospital setting. These include patients with acute kidney injury and patients treated with hemodialysis.

**Results:**

We used HCU to provide essential information about volume status which is otherwise not readily available. HCU allowed objective assessment of volume status, helping with clinical management of hospitalized patients and potentially avoiding harm.

**Conclusion:**

HCU can complement physical examination for volume assessment in hospitalized patients with acute kidney injury or those on hemodialysis. Our report highlights the need for systematic research in this area.

## 1 Introduction

Accurate assessment of volume and hemodynamic parameters are the crux of routine clinical practice. Patients with chronic kidney disease, acute kidney injury (AKI), cardiorenal syndrome, and those on dialysis comprise the majority of patients managed by general nephrology consultants and each requires accurate assessment of volume status for sound clinical decision-making. Inaccurate assessment of volume status can have significant consequences. Excessive diuresis or ultrafiltration lowers effective circulating volume leading to ischemic complications including AKI and delayed recovery from AKI as well as intradialytic hypotension, myocardial ischemia, and stunning in dialysis patients. Volume overload is also not inconsequential, contributing to cardiorenal syndrome, dyspnea, hypertension, and acute respiratory failure. Finding the right balance between iatrogenic volume depletion and inadequately treated volume overload remains a major clinical challenge.^[1,2]^

In the absence of objective data, even experienced clinicians disagree on subjective estimates of effective circulating volume.^[3–7]^ Body habitus, cardiac valvular dysfunction, or central lines often limit jugular venous pressure (JVP) assessment. Even with a keen ear, auscultation for rales or an S3 can be obscured by co-existing thoracic pathology. Similarly, although peripheral edema can often be readily seen, it does not necessarily reflect the state of the patient's effective circulating volume. These challenges of the bedside examination—which have no doubt plagued physicians for hundreds of years—may be alleviated by the routine use of the hand-carried ultrasound (HCU). Using HCU at bedside provides an opportunity for trained clinicians to collect objective information about a patient's volume status in a quick, low-cost, and noninvasive fashion thereby augmenting the standard physical examination in a powerful way.^[8]^ For example, HCU measurement of inferior vena cava (IVC) diameter and collapsibility can allow objective assessment of effective circulating volume.

At our institution, all internal medicine housestaff are trained to use HCU. We present the following selected case vignettes to demonstrate that objective bedside data on volume status obtained via HCU can help the renal consultant with clinical decision-making. The vignettes were collected retrospectively and patient consent was not feasible. The study was reviewed and approved with a waiver of consent by the Johns Hopkins Medicine Institutional Review Board.

## 2 Case vignettes

### 2.1 Cardiorenal syndrome: to diurese or not to diurese?

Mr AB was a 50-year-old with coronary artery disease, congestive heart failure, atrial fibrillation, diabetes, and chronic obstructive pulmonary disease, admitted with hypoxia and generalized edema. Initial attempts at diuresis with escalating doses of intravenous furosemide were not successful. His urine output remained low and his weight was unchanged over 8 days since admission (131 kg). A rising serum creatinine (increasing from 1.7 mg/dL on admission to 3.0 mg/dL) prompted renal consultation. On examination, he had soft pitting edema in both legs and elevated JVP. HCU evaluation of the IVC showed no collapsibility confirming expanded intravascular volume. We advised the team the AKI was most likely due to cardiorenal syndrome (type I) causing poor forward flow, and recommended further diuresis with inotropic support. A dobutamine infusion was administered with improvement in urine output and quick recovery of renal function back to baseline.

### 2.2 AKI and volume overload: to diurese or not to diurese?

Mr CD was a 60-year-old with nonischemic cardiomyopathy and diabetes admitted with progressively worsening dyspnea and lower extremity edema. We were consulted due to rising creatinine (from 1.9 to 2.7 mg/dL) in the setting of gentle diuresis (without change in weight). We noted that the patient had nephrotic-range proteinuria. Exam revealed extensive, soft pitting edema in both legs extending to the low back, along with elevated JVP. HCU of the IVC showed no collapsibility confirming clinical assessment of volume overload. We recommended intensifying the diuresis regimen with plans for a renal biopsy.

### 2.3 Dyspnea in a hemodialysis patient: increase ultrafiltration and lower dry weight?

Mr EF was a 59-year-old with congestive heart failure, aortic stenosis, diabetes, and end-stage renal disease on hemodialysis (target weight 65 kg), admitted with new-onset atrial fibrillation. On hospital day 5 (evening prior to dialysis), he developed increasing dyspnea thought to be due to volume overload. Although his weight was 63.2 kg the next morning (day 6 of hospitalization), the primary service was requesting extra ultrafiltration during routine hemodialysis with an attempt to lower dry weight. However, he had no peripheral edema on examination and lung examination did not reveal rales. HCU showed greater than 50% collapsibility of the IVC provideing objective confirmation of lack of volume overload. We avoided harm from extra ultrafiltration, which could have caused intradialytic hypotension. Further work-up revealed a recurrent right pleural effusion.

### 2.4 Intradialytic hypotension: decreased effective circulating volume?

Ms GH was a 56-year-old with chronic kidney disease stage 5, congestive heart failure, atrial fibrillation, and prior aortic and mitral valve replacements, admitted with volume overload and fatigue despite aggressive outpatient diuretic regimen with good adherence. We initiated hemodialysis due to refractory volume overload. With ultrafiltration her weight gradually improved from 127.2 to 123.1 kg after 4 hemodialysis session. During the 5th hemodialysis session, the patient became hypotensive with systolic blood pressure in the low 90s. Physical examination was still consistent with volume overload but the effective circulating volume was difficult to assess. HCU showed a distended, noncollapsible IVC providing objective data of volume expansion. We continued hemodialysis treatment with ultrafiltration without recurrence of hypotension and were gradually able to lower her weight to 121 kg.

### 2.5 Intradialytic hypotension and dyspnea: at target weight or not?

Mr IJ was a 68-year-old former smoker with severe emphysema, coronary artery disease, and end-stage renal disease with recent hemodialysis initiation, admitted with syncope after dialysis. His outpatient target weight was set at 66 kg. However, due to ongoing dyspnea, target weight was being lowered in the outpatient setting. He was orthostatic on admission and his weight was 63 kg. HCU of the IVC showed >50% collapsibility providing objective confirmation that he was not volume overloaded. We revised his target weight upwards and communicated it with the outpatient unit.

## 3 Discussion

These vignettes highlight HCU's capability of providing actionable diagnostic information at the bedside. By obtaining a more accurate and objective assessment of intravascular volume than can typically be gleaned from a traditional physical examination, we may see fewer delays in the initiation of appropriate management. Likewise, the risk of complications – such as intradialytic hypotension or AKI in the setting of inadvertent fluid removal – may also be better avoided.

Ultrasound technology is rapidly becoming miniaturized and available for real-time use at the point of care.^[9]^ Traditionally, ultrasound use was limited to subspecialists such as cardiologists. However, it is feasible to teach clinicians at various stages of training to adeptly and reliably use HCU for diagnostic and procedural tasks.^[10–12]^ At our own institution, all internal medicine housestaff rotate through a medicine wards team where they receive dedicated teaching and practice in using sonography to augment the cardiac examination and aid in volume assessment. We, and others, have previously reported that use of HCU can also be taught to hospitalists for the assessment of IVC collapsibility, left ventricular dysfunction, cardiomegaly, and pericardial effusion.^[13,14]^

**Figure 1:**
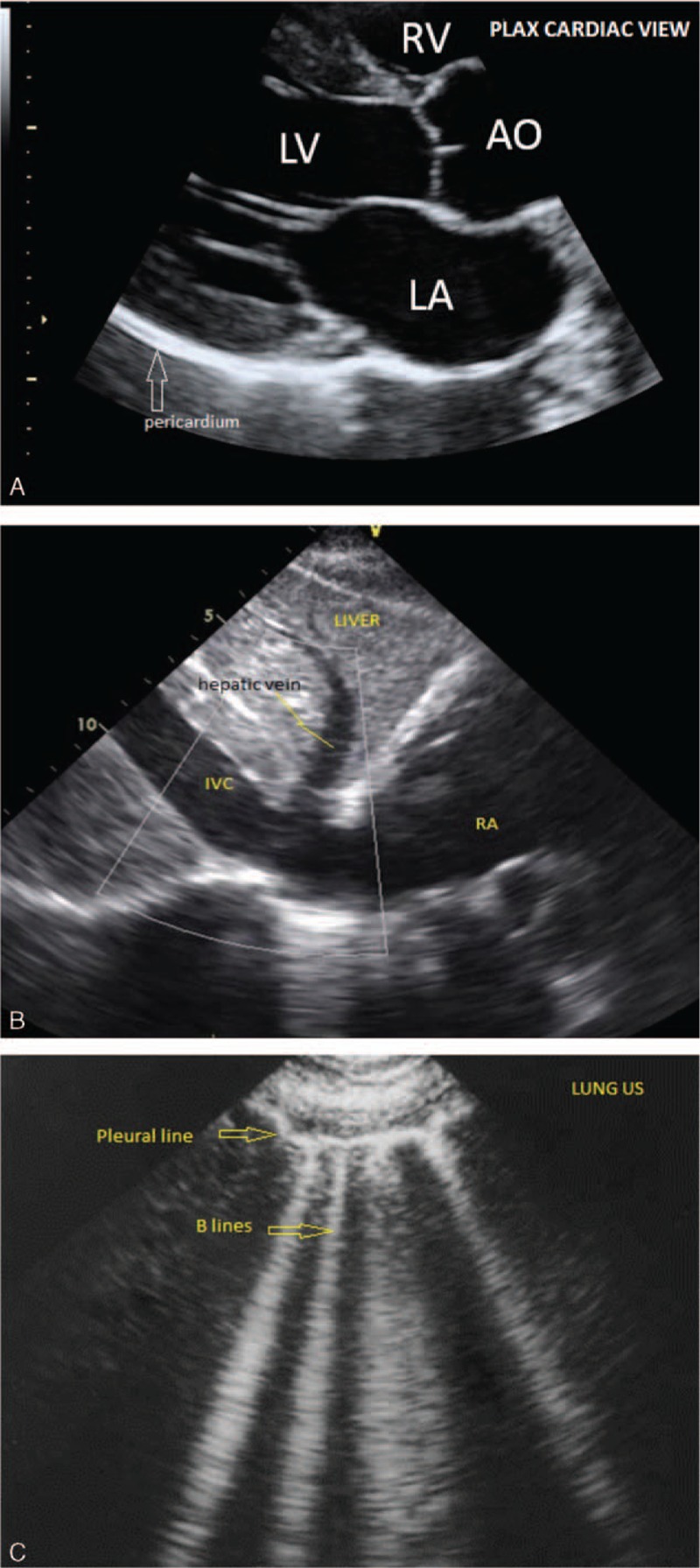
Images from hand-carried ultrasound obtained during point-of-care bedside use. (A) Cardiac parasternal long axis view demonstrating right ventricle (RV), left atrium (LA), left ventricle (LV) and aorta (AO). (B) Subxiphoid view demonstrating liver, hepatic vein, inferior vena cava (IVC), and right atrium (RA). (C) Lung view demonstrating pleura and “B” lines reflecting pulmonary edema.

HCU use specific to volume assessment in routine nephrology care is not widely utilized in the US. In the context of volume management, HCU can provide instantaneous information about pericardial or pleural effusions, pulmonary edema (left heart failure) and IVC diameter, and collapsibility (right heart failure) thereby enhancing personalized decision-making at the bedside (Fig. 1 and Table 1). Our vignettes suggest that HCU volume assessment may improve patient care. Direct visualization of the IVC in dialysis patients has the potential to not only assist in bedside diagnosis,^[15]^ but also allows the patient to see directly into his/her own body, thereby transforming the routine examination into a unique opportunity for education and connection between clinician and patient. Such an approach may improve patient's understanding of illness and improve self-efficacy and adherence. We believe that our case-vignettes are hypothesis generating, and we hope to stimulate formal educational and outcomes research using these tools.

**Table 1:**
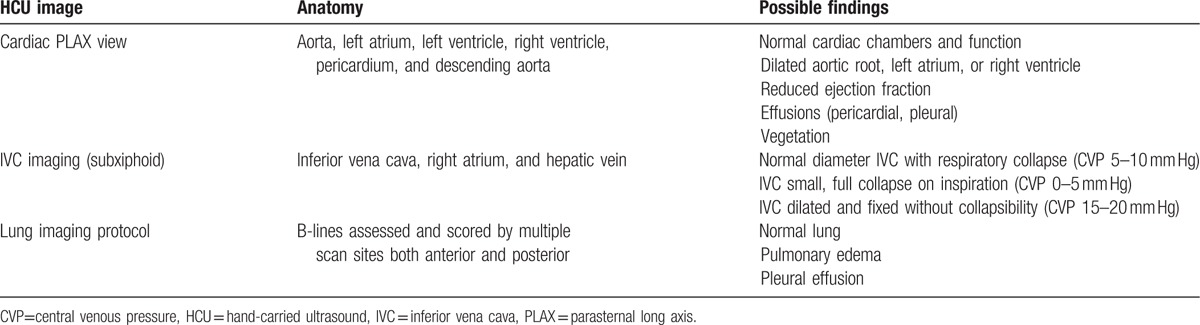
HCU for the assessment of volume status.

All new technology brings new challenges and limitations. We are optimistic about the potential benefits of routinely using ultrasound technology to assess volume status, however, as has been acknowledged elsewhere, if clinicians are not trained sufficiently or if time to properly examine patients is not available, diagnostic errors could result. It may also be undesirable to train clinicians on the use of yet another “gadget” which has the potential to take energy away from honing skills in the basics of patient assessment and physical diagnosis. However, immediate validation of clinical findings by bedside HCU may reinforce and improve physical examination skills.

It was 200 years ago when Laënnec's marvelous invention – the stethoscope – allowed physicians to enhance physical diagnosis by auscultation. We believe that HCU carries the same potential in 2016 as did the stethoscope in 1816. Clinicians and patients alike would benefit from taking a closer look at the risks, benefits and thoughtful use of HCU. Bedside volume assessment is one such specific application of this technology. Nephrologists, more than any other subspecialists, are at the forefront of volume assessment and can lead the way in assessing the role of HCU in volume-related, patient-centered outcomes.
